# Microbial hitchhiking: how *Streptomyces* spores are transported by motile soil bacteria

**DOI:** 10.1038/s41396-021-00952-8

**Published:** 2021-03-15

**Authors:** Alise R. Muok, Dennis Claessen, Ariane Briegel

**Affiliations:** 1 Institute for Biology, Leiden University, Leiden, The Netherlands; 2 Centre for Microbial Cell Biology, Leiden University, Leiden, The Netherlands

**Keywords:** Bacteria, Cellular microbiology

## Abstract

Streptomycetes are sessile bacteria that produce metabolites that impact the behavior of microbial communities. Emerging studies have demonstrated that *Streptomyces* spores are distributed through various mechanisms, but it remains unclear how spores are transported to their preferred microenvironments, such as plant roots. Here, we show that *Streptomyces* spores are capable of utilizing the motility machinery of other soil bacteria. Motility assays and microscopy studies reveal that *Streptomyces* spores are transported to plant tissues by interacting directly with the flagella of both gram-positive and gram-negative bacteria. Genetics experiments demonstrate that this form of motility is facilitated by structural proteins on the spore coat. These results demonstrate that nonmotile bacteria are capable of utilizing the motility machinery of other microbes to complete necessary stages of their lifecycle.

## Introduction

Bacteria belonging to the genus *Streptomyces* are an integral component of diverse ecosystems and are well-known to produce chemically diverse metabolites, including the vast majority of all clinically relevant antibiotics [1, 2]. Soil-dwelling Streptomycetes, such as *Streptomyces coelicolor* (*Sc*), colonize plant roots and provide the associated plant protection from potential phytopathogens through antibiotic secretion [1]. The symbiosis of Streptomycetes with their plant hosts has been shown to improve plant health and productivity, and thereby provides a potential sustainable solution to increase crop yields [2–5]. The lifecycle of Streptomycetes is complex and involves stages of aerial hyphae formation on the soil surface to produce spores, and spore germination on plant roots to produce filamentous colonies [1]. Immotile *Streptomyces* bacteria distribute their spores over long distances through attachment to insects and nematodes, but it is unclear how they relocate over short distances to their preferred microenvironments such as plant root systems [6, 7].

While *Streptomyces* are nonmotile, many other soil microbes, such as *Bacillus subtilis* (*Bs*) and *Pseudomonas fluorescens* (*Pf*), are motile and can move through their environment by regulating the rotation of flagella [8]. On solid surfaces, flagellar rotation enables cell swarming. During swarming, cells are densely packed and continuously move outward toward unoccupied areas. In liquid media, flagella enable cell swimming where the cells move independently and can rapidly change swimming direction. In addition to flagellar motility, some bacteria can also move through a passive diffusion on surfaces called sliding [8]. Sliding does not involve flagella but occurs when cells are pushed through the forces generated by the outward growing colony.

Recent reports have revealed that microbe transport by inter-species interactions can occur between motile and immotile microbes [9]. These studies demonstrate that inter-microbial transport occurs among organisms natively found on abiotic surfaces [10, 11], plant surfaces [12], and within the soil [13, 14]. In some instances, the transportation of human pathogens are facilitated on abiotic surfaces, including nonmotile Staphylococcal species that directly adhere to their mobile partners [10], *Aspergillus fumigatus* (*Af*) spores that interact with the flagella of motile bacteria [14], various nonmotile human microbiome bacteria that are carried by *Capnocytophaga gingivalis* [15], and *Legionella pneumophila* that is transported internally by their amoebae hosts [16].

Here, we demonstrate that spores of the sessile Streptomycetes, such as *Sc*, are transported by *Bs* to their preferred microenvironment. *Sc* and *Bs* are both soil-dwelling bacteria that utilize plant root exudate as a nutritive source [1, 17]. Using microscopy methods, motility assays, and genetics approaches, we demonstrate that *Bs* transports *Sc* spores via direct attachment to *Bs* flagella, a mode of transportation we call “hitchhiking”. Hitchhiking is dependent on the conserved rodlin proteins, which form a fibrous outer layer on the spore coat of almost all Streptomycetes, but with a hitherto unclear function [18, 19]. These results exemplify that nonmotile bacteria are capable of utilizing the motility machinery of other microbes to occupy advantageous environments, and that this mode of transport may be widespread in nature.

## Results

### *Bacillus subtilis* disperses *Streptomyces coelicolor* spores

**Fig. 1:**
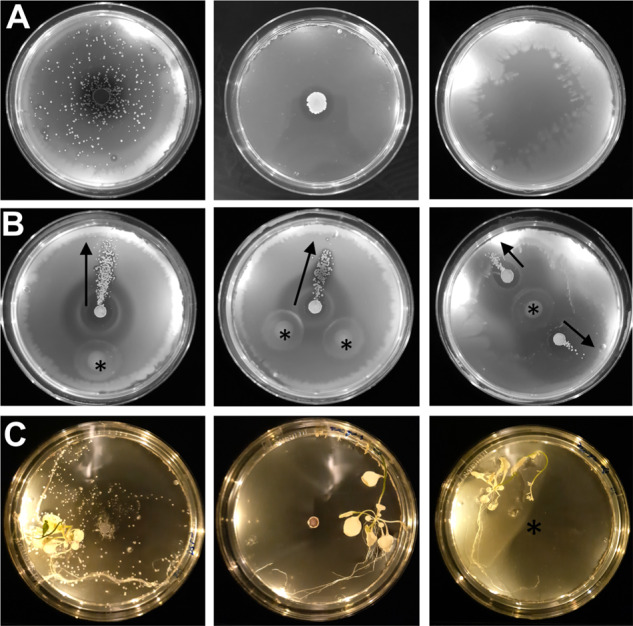
*S. coelicolor* spores are transported by *B. subtilis*. **A** When *Sc* and *Bs* are innoculated on the center of a swarm plate, visible *Sc* colonies (white dots) are apparent and are only dispersed in the presence of motile *Bs*. Left: *Sc* with *Bs*. Middle: *Sc* alone. Right: *Bs* alone. **B** When *Sc* and *Bs* are innoculated in different positions on swarm plates, the *Sc* colonies are dispersed in the swarming direction of the *Bs* cells (black arrows). Asterisks denote the *Bs* innoculation sites. **C** *Bs* moves spores toward plant tissues. Left: *Sc* with *Bs*. Middle: *Sc* alone. Right: *Bs* alone, asterisk denotes the *Bs* innoculation site.

Transportation of *Sc* spores by *Bs* was demonstrated by mixing isolated *Sc* spores with a liquid culture of *Bs* followed by inoculation onto an agar swarm plate and incubation. After 5 days, *Sc* colonies are visible on the plate and are only dispersed in the presence of *Bs* in all samples tested (*n* = 13) (Fig. 1A). Spore dispersal by *Bs* occurs across the entire surface of the plate and to the plate’s edge (4.5 cm from the inoculation point). To demonstrate that the *Sc* spores are being moved by *Bs* cells and not merely “floating” in the expanding *Bs* colony, we conducted identical assays but inoculated the *Sc* spores and *Bs* culture separately and onto different areas of the plate. The resulting *Sc* colonies form streaks across the plate that emanate from the *Bs* inoculation site in a predictable manner in all samples tested (*n* = 10) (Fig. 1B). This experiment was repeated on 12 cm plates and demonstrate that *Sc* spores are dispersed to the edge of the plate, which is 10 cm from the spore innoculation point (*n* = 3) (Fig. S1A). To determine the effeciency of transport, this experiment was conducted with dilutions of *Sc* spores so that the total number of *Sc* colonies would germinate without overlap and could be individually counted. The results demonstrate that 86 ± 5.6% of the apparent colonies are located outside of the initial innoculation point under these conditions (*n* = 4) (Fig. S1B).

### *B. subtilis* transports *S. coelicolor* spores to plant tissues

In nature, *Sc* and *Bs* thrive near plant roots that excrete exudates but only *Bs* can move toward the root systems. We conducted assays to determine if *Bs* can transport *Sc* spores to plant tissues. Assays with the *Bs* strain alone demonstrate that the plates become “cloudy” with *Bs* cells in areas around plant tissues, perhaps due to the presence of nutritive plant exudates that facilitates bacterial growth (Fig. 1C). Like previous experiments, the *Sc* spores alone do not exhibit movement unless they are co-inoculated with *Bs* cells, and the dispersed spores preferentially establish colonies near plant tissues in all instances (*n* = 5) (Fig. 1C).

### Spore dispersal occurs with swarming and sliding *Bs* strains

**Fig. 2:**
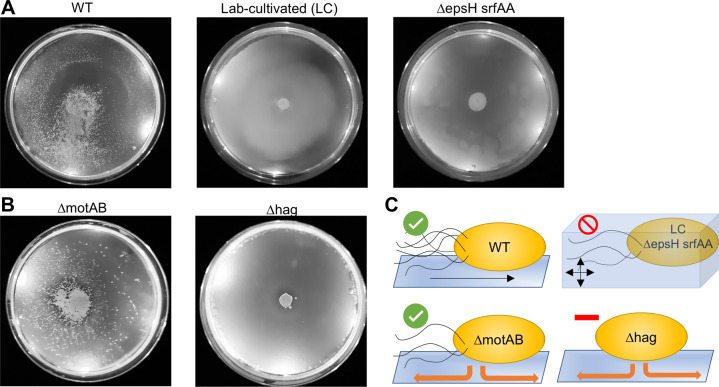
*B. subtilis* can transport spores via swarming and sliding. **A** The WT (swarming strain) can transport spores on agar plates (0.27–0.5%) (*n* = 13), but swimming only strains (laboratory-cultivated WT strain or ΔepsH srfAA) cannot (0.25–0.3% agar) (*n* = 6 and *n* = 8, respectively). Quantification of the results is shown in Fig. S2. **B** A *Bs* ΔmotAB strain that possesses flagella but lacks flagellar motility can disperse spores via sliding at WT levels (*n* = 6). A *Bs* Δhag strain that does not possess flagella cannot disperse the spores via sliding at WT levels (*n* = 6). Quantification of results is shown in Fig. S2. All Δhag plates are shown in Fig. S3. **C** In summation, spores are dispersed by swarming (WT) and sliding (ΔmotAB) *Bs* cells in a flagella-dependent manner. However, spores are not dispersed via swimming (lab-cultivated or ΔepsH srfAA), and are significantly less dispersed via sliding in the absence of flagella (Δhag).

*Bs* has two modes of flagellar-mediated motility, swimming and swarming, that occur in liquid environments and on solid surfaces, respectively. When *Bs* senses that it is on a solid surface, it will differentiate into a swarmer cell that has a significant increase in the number of flagella and produces hydrophobic surfactants, such as surfactin, to efficiently spread across the surface [20–22]. In our experiments, we utilized an undomesticated strain of *Bs* (NCIB3610) that can swarm, unlike common laboratory strains that lack the ability to differentiate into swarmer cells and fail to produce surfactin to undergo swarming motility (Table S1) [20, 22–24]. To determine if the *Sc* spores are transported by both swarming and swimming motilities, we repeated the experiments with a laboratory-cultivated *Bs* strain that is incapable of swarming, and spore transport does not occur on the agar swimming plates (0.27% agar) (Figs. 2A and S2) [20]. Importantly, the laboratory-cultivated strain has accumulated many genetic defects in addition to defective swarming capabilities. To ensure that the decrease in spore dispersal can be attributed to limitations of bacterial swimming, we utilized a *Bs* strain with an undomesticated genetic background but has been genetically altered so that it is only capable of swimming motilities (Δ*epsh srfAA*, DK1484). Like the laboratory-cultivated strain, this strain does not disperse *Sc* spores on swim plates (Figs. 2A and S2). Therefore, we conclude that spore transport can be accomplished by swarming motility but not swimming motility.

In addition to flagellar motilities, *Bs* can also move through sliding on surfaces. To determine if spores can be dispersed through sliding, we utilized a *Bs* mutant that has flagella but the flagella cannot rotate (Δ*motAB*, DS222) [25], and a mutant that does not possess flagella (Δ*hag*, DS1677) [26]. The Δ*motAB* strain moves spores over the entire surface and to the edge of the agar plates in all samples (4.5 cm from the inoculation point) (*n* = 6) (Figs. 2B and S2). The number of dispersed spores cannot be ascertained due to overlapping colonies that result in apparent smears across the plates, but each plate contains over 100 dispersed *Sc* colonies. The Δhag strain shows severely reduced dispersal, where an average of 3.2 spores are transported with an average maximum distance of 0.95 ± 0.85 cm from the inoculation point (*n* = 6) (Figs. 2B, S2 and S3). Therefore, we surmise that spore dispersal can also occur through sliding but is facilitated by the presence of flagella (Fig. 2C). As an additional control, we also conducted these experiments by first spreading the *Bs* cells across the surface of the plate and then inoculating the *Sc* spores in the center. Spore dispersal was significantly reduced for both the *Bs* WT (2.06 ± 0.05 cm) and Δ*motAB* (1.25 ± 0.16 cm) strains when compared to co-inoculation in the center of the plate (Figs. S2 and S4). These data indicate that the *Sc* spores are transported by *Bs* cells that continuously move away from the inoculation point and are not transported after the motile *Bs* has covered the surface of the plate.

### *S. coelicolor* spores attach to *B. subtilis* flagella

**Fig. 3:**
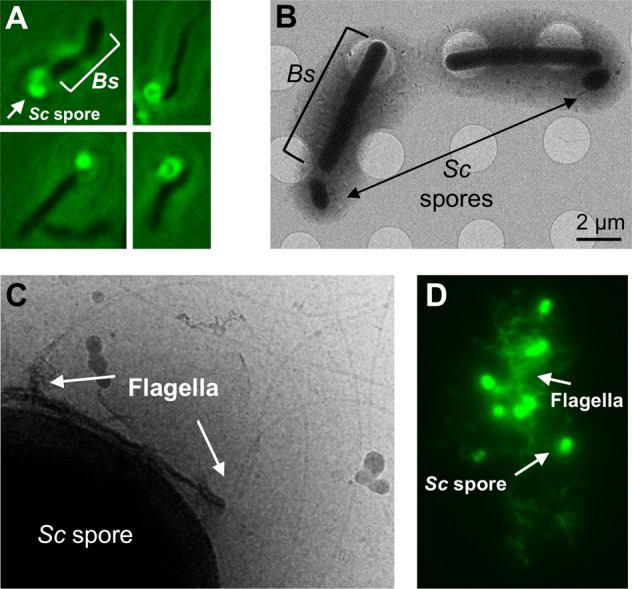
Microscopy methods indicate that *Sc* spores directly adhere to *Bs* flagella. **A** Fluorescence microscopy of dye-labeled spores with unlabeled *Bs* cells demonstrate that the spores localize to the cell poles of *Bs*. **B** Cryo-electron microscopy samples of mixed *Sc* spores and *Bs* cells reveal that the spores do not directly adhere to the *Bs* cell body. **C** Cryo-EM shows the *Bs* flagella colocalize with the *Sc* spore coat. **D** Fluorescence microscopy of dye-labeled sheared flagella and spores demonstrate that spores directly interact with *Bs* flagella to form extended associations of both components.

We utilized several microscopy methods to elucidate a mechanism for *Sc* spore dispersal by *Bs*. Fluorescently labeled *Sc* spores were imaged under a fluorescence microscope and were immotile as expected (Supplementary Movie 1). However, when *Bs* cells were added to the fluorescent spores, the spores localize near the *Bs* cell poles (Supplementary Movie 2 and Fig. 3A). In some instances, the spores are stationary on the surface of the glass slide and an associated *Bs* cell is seen rotating around the spore (Movie 1). The observed *Bs* cell rotation in these assays is reminiscent of rotations seen in *Bs* cells that have their flagella chemically tethered to a solid surface, whereby the torque generated by the immobilized flagella induces rotation of the cell body [27]. This observation suggests that the *Sc* spores adhere directly to the *Bs* flagella, and therefore effectively mimic a flagellar tether in these instances. To verify that the *Sc* spores do not directly interact with the *Bs* cell body, we imaged a mixture of *Sc* spores and *Bs* cells with a cryo-electron microscope. Like the fluorescence microscopy images, the *Sc* spores are localized near the *Bs* cell poles but do not make direct contact with the *Bs* cell body (Figs. 3B and S5A). In total, ~77% of the spores are located within 1 μm of a *Bs* cell poll (*n* = 35 spores). To determine if the spores interact with *Bs* flagella, we utilized a *Bs* “mini-cell” strain (*minD*::TnYLB) that lacks the excreted material inhibiting their direct visualization. Indeed, when mixed with *Sc* spores, the flagella can be seen co-localizing with the spores in two-dimensional cryo-EM images (Figs. 3C and S5B).

*Sc* spore adherence to *Bs* flagella was further confirmed by visualization of a mixture of *Sc* spores with sheared flagella via fluorescence microscopy. Fluorescent labeling of *Bs* flagella was accomplished using a *Bs* strain (DS1919) that is mutated in a single flagellin residue (T209C) [28]. The surface-exposed thiol allows for direct labeling with dyes that possess a reactive maleimide group. This dye also labels proteins on the surface of the *Sc* spore coat. Indeed, when sheared *Bs* flagella are isolated and mixed with spores, and unassociated flagella are washed from the mixture, a majority of spores still retain associated flagella. In total, ~64% of the spores are associated with flagella (*n* = 130 spores). In some instances, large “clumps” of spores entangled in flagella are observed (Figs. 3D and S5C). In comparison, the flagella without spores added do not form aggregates and are randomly dispersed (Fig. S5C).

### Hitchhiking is conserved in Streptomycetes

**Fig. 4:**
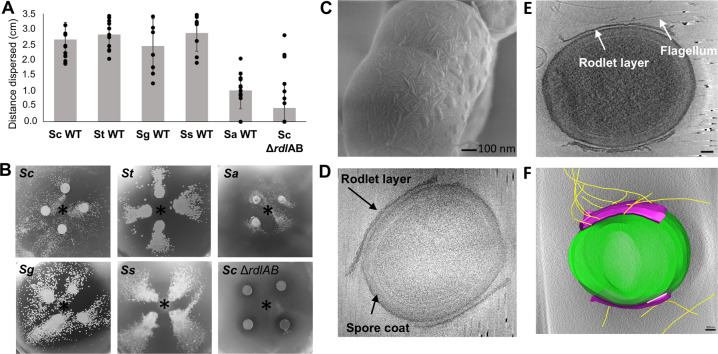
Hitchhiking of *Streptomyces* spores is facilitated by the presence of rodlin proteins. **A***Bs* swarm assays with both wild-type (WT) *Streptomyces* spores (*S. coelicolor n* = 20*, S. tendae n* = 12*, S. griseus n* = 8, *S. scabies n* = 8, *S. avermitilis n* = 12) and *Sc* spores lacking the rodlin proteins (*Sc* Δ*rdl*AB *n* = 24) demonstrate that spores are dispersed in all tested WT species but with *S. avermitilis* dispersed the shortest distance, and dispersal is abrogated by the loss of the rodlins in *Sc*. Results are expressed as the mean of dispersal distance ± standard error of the mean. Differences for *Sc* Δ*rdl*AB and *Sa* dispersal compared to WT *Sc, St, Sg*, and *Ss* strains are statistically significant using a two-tailed null hypothesis significance test (*p* < 0.05). **B** Representative images of *Bs/*spore swarm plates from **A**. The *Bs* innoculation site is denoted with an asterisk. **C** SEM of WT *Sc* spores shows the rodlet layer with pairwise rodlets of 20 nm spacing. **D** A representative cryo-ET image of isolated *Sc* WT spores shows that the rodlet layer does not cover the entire spore but leaves the poles exposed (*n* = 22). **E** Cryo-ET reconstructions show that flagella preferentially interact with the rodlet layer (*n* = 12). Scale bar 100 nm. **F** Segmentation of the reconstruction from **E** clearly demonstrates the flagella:rodlin interaction. Spore body: green, rodlet layer: purple, flagella: yellow. Scale bar 100 nm (Color figure online).

To determine if spore dispersal by *Bs* also occurs in other *Streptomyces* species, we conducted *Bs* swarm plate assays with *Streptomyces tendae* (*St*), *Streptomyces griseus* (*Sg*)*, Streptomyces scabies* (*Ss*), and *Streptomyces avermitilis* (*Sa*). To quantify spore dispersal, we prepared swarm plate assays where *Bs* cells were inoculated at the center of the plate (9 cm in diameter) and the isolated spores are inoculated in four equidistant positions around the *Bs* inoculation site. The maximum dispersal distance, which is the distance from the center of the spore inoculation site to the most distant dispersed colony, was measured for each of the four spore samples. The wild-type (WT) *Sc* spores are dispersed by *Bs* in 100% of samples and are moved an average maximum distance of 2.67 ± 0.43 cm from the initial inoculation point (*n* = 20). Likewise, these assays demonstrate that *St* (*n* = 12)*, Sg* (*n* = 8), and *Ss* (*n* = 8) spores are dispersed in 100% of samples to similar distances as WT *Sc* spores. However, the *Sa* spores are dispersed at significantly shorter distances (*n* = 12) (Fig. 4A, B) and are dispersed in 83% of the samples. As the last common ancestor of *Sc* and *Sg* existed more than 200 million years ago and both species are capable of hitchhiking, these data suggest that the ancestor also possessed this dispersal mechanism and it remained conserved.

### Spore dispersal by *B. subtilis* is facilitated by the rodlin proteins

The outer surface of most *Streptomyces* spores is characterized by a fibrillar rodlet layer, which is a striated pattern of pairwise aligned rodlets composed of the rodlin proteins [18, 19]. Scanning electron microscopy (SEM) images of *Sc* and *Ss* spores show the striated rodlet layer (Fig. 4C). In previous studies, the rodlets of *Sc, S. lividans, St, Sg, and Ss* were visually indistinguishable [19, 29]. Using electron microscopy images from this and previous studies, we measured the spacing of the rodlets in these species, which is highly conserved and around ~20 nm (when measured from the center of the rodlet fibers) (Table S2). Furthermore, the rodlin proteins from *Sc, St*, and *Sg* have ~34% sequence identity despite the species’ distant evolutionary relation (Fig. S6) [19].

Intriguingly, *Sa* spores are less widely dispersed than the other *Streptomyces* species and it is the only tested species that natively lacks rodlin proteins [19]. In agreement, an *Sc* mutant strain that lacks the rodlin proteins (Δ*rdlAB*) abrogates hitchhiking by *Bs* (*n* = 3, Figs. S2 and S7). Importantly, previous studies demonstrate that the *Sc* Δ*rdlAB* strain is not delayed in germination, and does not exhibit any behavioral or phenotypic change compared to the WT strain with the exception of the rodlet layer [18, 19, 30]. In contrast, *Sc* mutants that lack proteins which produce polysaccharides found on the surface of Streptomycetes (Δ*cslA* and Δ*matAB*) are unaffected (*n* = 3, Figs. S2 and S7) [31]. However, spore dispersal was not completely abolished in the Δ*rdlAB* strain. Using identical swarm plate assays described in the section above, the Δ*rdlAB* strain is dispersed with an average maximum distance of 0.46 ± 0.82 cm from the initial inoculation point and dispersal occurs in 33% of the samples (*n* = 24) (Fig. 2).

To characterize how rodlins interact with flagella in three dimensions, we conducted cryo-ET experiments of samples containing *Bs* minicells and *Sc* spores. Reconstructions show that the *Sc* spores are oval shaped and possess a thick coat. The rodlet layer can be seen as a sheath around the lateral sides of the spore with frayed edges, leaving the poles exposed, and suggest that the rodlet sheath easily peels away from the cell body (*n* = 22) (Fig. 4D). A similar spore morphology has also been observed in *Streptomyces albus* [32]*. Bs* flagella accumulate around and directly interact with the rodlet layer (*n* = 12) (Fig. 4E, F and Movie 2). However, due to the thickness of the spores the resolution is limited and we could not deduce if the flagella preferentially bind specific features of the rodlet layer. Collectively, these data suggest that the rodlet layer facilitates spore dispersal by interacting directly with flagella.

### Hitchhiking of *S. coelicolor* spores is not limited to *Bacillus*

**Fig. 5:**
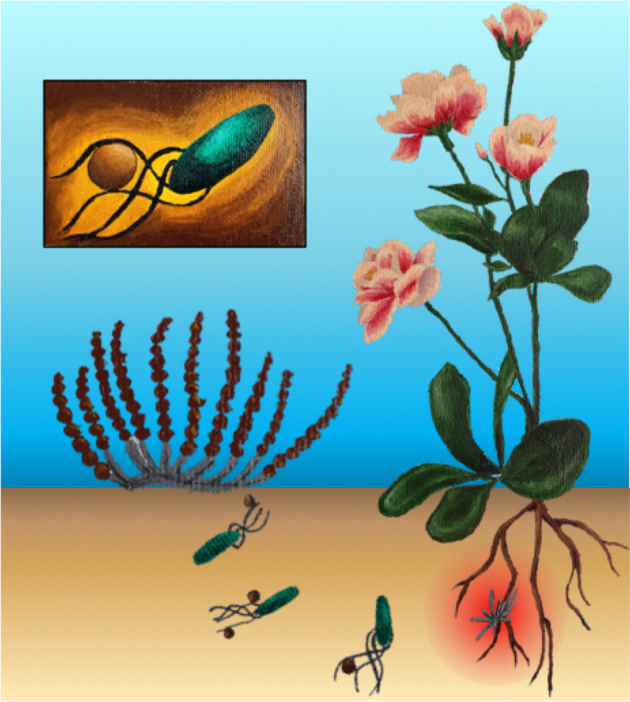
An overview of the hitchhiking model. Aerial *Streptomyces* spores are transported on the cm-scale to plant root systems by directly adhering to the flagella of motile bacteria (inset). Here, the spores germinate and produce antibiotics (red gradient) to ward off microbial competitors (Color figure online).

Although *B. subtilis* is ubiquitous in soil, other genera are also flagellated and may also contribute to dispersal of *Sc* spores. We therefore conducted swarm plate assays with a *P. fluorescens* WT strain (R1SS101), which is also natively associated with plant roots [33]. Importantly, *Pf* disperses spore similarly to *Bs*, but *Sc* colonies appear in patterns that are reminiscent of *Pseudomonas* swarm patterns [34] (*n* = 6) (Fig. S8). These data demonstrate that hitchhiking is a widespread mechanism that allows *Streptomyces* spores to disperse at cm-scales (Fig. 5).

## Discussion

Sessile Streptomycetes have a complex lifecycle that involves formation of aerial hyphae that differentiate into spores. The spores of some *Streptomyces* species, including *Sc*, are dispersed over long distances by direct attachment to insects and nematodes [6]. Intriguingly, recent reports identify that specific volatile metabolites secreted by Streptomycetes attract arthropods as a mechanism for spore dispersal [7], and they can induce the formation of *Streptomyces* “explorer cells”, while simultaneously starving microbial competitors [35]. However, it is unclear how the spores are dispersed specifically at the centimeter scale to plant root microenvironments. Here, we demonstrate that *Streptomyces* spores are able to utilize the motility machinery of motile soil microbes by directly attaching to their flagella. While these experiments demonstrate that *Sc* spores are dispersed by *Bs* and *Pf* regardless of their destination, these motile bacteria are also known to associate with plant roots. Therefore, this mechanism of dispersal, called hitchhiking, may provide *Streptomyces* spores a mechanism for translocation to beneficial environments. Indeed, assays with *A. thaliana* plants demonstrate that *Bs* can transport *Sc* spores to plant tissues. This allows spores to germinate near nutrient-rich plant exudate to generate filamentous colonies that produce antibiotics, thereby protecting the plant from potential phytopathogens.

Hitchhiking is facilitated by two spore coat proteins, RdlA and RdlB, which are conserved in most Streptomycetes. These proteins assemble into pairwise aligned filaments, called rodlets, on the outer surface of the spores and are spaced ~20 nm apart (Table S2). Until now, the function of the rodlets has remained elusive [30]. Interestingly, the diameter of the bacterial flagellar filament is also ~20 nm [22, 36]. Therefore, it is possible that the rodlet layer provides a gripped surface for the flagella, which become “wrapped” in the grooves made by the rodlin proteins and thereby facilitates spore transport. However, our cryo-ET data can not support such speculations due to the limited resolution of the whole-cell reconstructions. Therefore, it is still unclear what properties of the rodlet layer encourage interactions with flagella.

Emerging studies have demonstrated that flagella preferentially interact with hydrophobic surfaces and flagellin can undergo methylation to increase flagella hydrophobicity [37–39]. This increase in hydrophobicity allows pathogenic bacteria to adhere to host cells [37–39], and flagellar adherence to plant cells has also been implicated in establishing colonization [40, 41]. Flagella hydrophobicity may facilitate interactions with hydrophobic spores and may account for spore transport that is seen in the absence of rodlins, given that the spore surface without rodlins remains hydrophobic (in *Sa* WT and *Sc* Δ*rdl*AB strains) [18, 42, 43]. Therefore, the same flagellar interactions that facilitate adherence to plant roots may also contribute to adherence to spores, and necessitate that motile bacteria not evolve to eliminate this interaction. Since spore:flagella interactions are in-part facilitated by hydrophobic interactions, this mode of transport may be influenced by environmental factors that impact Van der Waals screening distances such as salt concentrations and pH.

Although spore hitchhiking seems disadvantageous to the motile partner, previous research has shown that fungal hyphae can form so-called “fungal highways” that serve as bridges for motile bacteria over air gaps (such as air gaps found in soil) [14, 44, 45]. Like fungi, Steptomycetes form aerial hyphae that are structurally similar to fungal hyphae [46]. Therefore, Streptomycetes may be able to form “bacterial bridges” for their motile partner, but such interactions have not been reported yet. If such “bacterial bridges” do form, this would supply the system with a synergistic transport that has been previously observed between nonmotile fungal spores and motile bacteria [14].

The hitchhiking model is supported by a previous study that examines the interaction of two other root-colonizing microbes: the immotile fungus *Af* and the motile bacterium *Paenibacillus vortex* (*Pv*) [14]. *Af* spores are demonstrated to be dispersed by *Pv* in a swarming-dependent manner via direct attachment to flagella; dispersal is abrogated by the addition of excess purified *Pv* flagella or perturbations to the *Af* spore coat [14]. Furthermore, scanning EM micrographs show direct contact between *Pv* flagella and *Af* spores [14]. Although this study does not identify the spore coat component(s) responsible for adherence to flagella, *Aspergillus* spores also possess a rodlet layer [47]. Additionally, this study demonstrates that some *Penicillium* species are also transported by *Pv*, and these fungi possess a rodlet layer [48]. Collectively, these data may suggest that hitchhiking of spores onto motile bacteria via the formation of a striated rodlet layer is a dispersal mechanism that convergently evolved in both domains of life.

The colonization of plant roots by some Streptomycetes improves plant health and performance in a natural and sustainable manner [2–5]. Therefore, our data are applicable to industrial initiatives that aim to improve soil conditions for *Streptomyces* root colonization. Likewise, many *Aspergillus* fungi, like *Af* and *A. niger*, are human and plant pathogens. Therefore, insights into hitchhiking of these sessile organisms may elucidate unknown infection mechanisms.

## Methods and materials

### *Streptomyces* spore isolation

The following *Streptomyces* strains were used in this study: *S. coelicolor* M145 [49], *S. coelicolor* Δ*rdlAB6* [42], *S. tendae* Tü901/8c [50], *S. griseus* (ATCC 13273), *S. avermitilis* (ATCC 31267), and *S. scabies* ISP5078. Spores were harvested from MS agar plates and quantified as described before [49].

### *Bacillus subtilis* cultivation

The undomesticated *Bs* strain NCIB3610 [23] 25% glycerol stock was placed into 5 ml of LB and grown overnight at 30 °C. After 16 h of growth, 100 μl of the overnight culture was diluted into 5 ml of LB and grown at 37 °C to an O.D. of 0.4–0.5.

### *Pseudomonas fluorescens* cultivation

The *Pf* strain R1SS101 25% glycerol stock was placed into 5 ml of 50% TB and grown overnight at 30 °C. After 16 h of growth, 100 μl of the overnight culture was diluted into 5 ml of 50% TB and grown at 37 °C to an O.D. of 0.4–0.5.

### Swarm and swim plate assays

Swarm and swim plates were conducted on nutrient broth plates (0.5% peptone, 0.3% yeast extract, 0.5% NaCl) containing specific amounts of agar (0.27–0.5%). All components were mixed, autoclaved for 20 min, and 30 ml of the media was poured into a plastic petri dish with a 9 cm diameter. The plates were cooled for 30 min in a sterile fume hood and then stored in a 4 °C fridge for a maximum of 1 week. To determine if *Sc* spores are dispersed by *Bs*, 3 μl of *Bs* cells are inoculated onto the plate and 3 μl of *Sc* spore stocks are either added on the *Bs* inoculation site or to a separate inoculation site. The plates are incubated at 30 °C for 5 days and imaged on a light box.

For plates that first had *Bs* cells spread across the surface, 100 μl of *Bs* cells at an O.D. 0.4–0.5 were pipetted onto the plate (0.5% agar) and then spread over the surface of the plate using an L-spreader. Then 3 μl of *Sc* spore stock was added to the center of the plate. Plates were incubated at 30 °C for 5 days and imaged on a light box.

For all plate images, the distance of spore dispersal was determined using Image J bundled with Java 1.8.0_172 software. To measure the distances, the diameter of the petri dish (9 cm) was used as a reference scale. Then, a line was drawn from the inoculation point of the spores to the *Streptomyces* colony that is furthest from the inoculation point. The distance of the line was calculated based on the value of the reference scale.

### Fluorescence microscopy

For fluorescent labeling of spores with unlabeled *B. subtilis*, 10 μl of *Sc* spore stock was added to 1 ml of iced LB. The 1 μl of the fluorescent styryl dye, FM2–10 (Thermo Fisher Scientific), was added to the 1 ml solution and inverted to mix. Excess dye was removed by rinsing the spores 4X with 1 ml of iced LB via centrifugation and decanting. After the final decantation, 1 ml of *Bs* cells with an O.D. of 0.4 were added to the spores, mixed via pipetting, and incubated at ambient temperatures for 5 min. Immediately before imaging, 5 μl of the samples were placed on a glass slide and a glass coverslip was placed on top. The sample was imaged on a Zeiss Axioscope A1 fluorescent microscope scope equipped with an Axiocam Mrc5 camera (Zeiss) in the Institute of Biology Microscopy Unit using a GFP filter. Images were collected and processed using Axiovision software (Zeiss).

For fluorescent labeling of sheared *B. subtilis* flagella with spores, *B. subtilis* (strain Δhag amyE::Phag-hagT209C spec) was grown to an O.D. of 0.6, and 2 ml of the cells were pelleted via centrifugation and resuspended in 1 ml of PBS buffer pH 7.5. The 1 ml suspension was passed back-and-forth between two 5 ml syringes with 21 gauge needles that were connected by a plastic tube (10 cm long with an inner diameter of 0.58 mm). The cell suspension was gently passed back and forth between the syringes 50 times, with 1 min pauses every ten passes. The cells were removed from the mixture via centrifugation at 5000 × *g* for 5 min. The supernatant containing the flagella were then centrifuged once again to remove any residual cells. In total, 5 μg/ml Alexa Fluor 488 C_5_ maleimide dye was added to the suspension and incubated for 5 min at room temperature. In total, 10 μl of *Sc* WT spore stock was then added to the mixture. The tube was gently inverted 50 times and the spores with associated flagella were pelleted via centrifugation at 5000 × *g* for 5 min. The pellet was washed with 1 ml PBS buffer pH 7.5 and centrifuged once again. The resulting pellet was resuspended in 50 μl PBS buffer pH 7.5. The sample was imaged on a Zeiss Axioscope A1 fluorescent microscope scope equipped with an Axiocam Mrc5 camera (Zeiss) in the Institute of Biology Microscopy Unit using a GFP filter. Images were collected and processed using Axiovision software (Zeiss).

### Cryo-electron microscopy

*B. subtilis* cells were grown to an O.D. of 0.5 and 1 ml of *B. subtilis* cells were mixed with 5 μl *S. coelicolor* spores glycerol stock and incubated at ambient temperatures for 5 min. Cells were concentrated by centrifugation and 3 μl aliquots of the cell suspension are applied to glow-discharged R2/2 200 mesh copper Quantifoil grids (Quantifoil Micro Tools), the sample was pre-blotted for 30 s, and then blotted for 2 s. Grids were pre-blotted and blotted at 20 °C and at 95% humidity. The grids were plunge frozen in liquid ethane using an automated Leica EM GP system (Leica Microsystems) and stored in liquid nitrogen. The grids were imaged on a 120 kV Talos L120C cryo-electron microscope (Thermo Fisher Scientific) at the Netherlands Center for Electron Nanoscopy.

### Cryo-electron tomography

*B. subtilis* mini-cell strain was grown from a 20% glycerol stock to an O.D. of 0.6 in 50 ml of LB. The cells were centrifuged at 8000 × *g* for 30 min. The supernatant was collected and then centrifuged at 12,000 × *g* for 20 min. The resulting cell pellet was resuspended in 20 μl of LB and 8 μl of WT *Sc* spore stock was added to the cell mixture. A 1/10 dilution of protein A- treated 10-nm colloidal gold solution (Cell Microscopy Core, Utrecht University, Utrecht, The Netherlands) was added to the mixture and mixed by pipetting. The grids were prepared using an automated Leica EM GP system (Leica Microsystems) with the sample chamber set at 20 °C and at 95% humidity. In total, 3 μl of the sample mixture was applied to a freshly glow-discharged copper R2/2 200 grid (Quantifoil Micro Tools), pre-blotted for 30 s, and then blotted for 2 s. The grid was plunge frozen in liquid ethane and stored in liquid nitrogen.

Images were recorded with a Gatan K3 Summit direct electron detector equipped with a Gatan GIF Quantum energy filter with a slit width of 20 eV. Images were taken at a magnification of ×19,500, which corresponds to a pixel size of 4.4 Å. Tilt series were collected using SerialEM with a bidirectional dose-symmetric tilt scheme (−60° to 60°, starting from 0°) with a 2° increment. The defocus was set to −12 μm and the cumulative exposure per tilt series was 160 e-/A^2^. Bead tracking-based tilt series alignment and drift correcting were done using IMOD [51] and CTFplotter was used for contrast transfer function determination and correction [52]. Tomograms were reconstructed using simultaneous iterative reconstruction with iteration set to 4. Segmentation was done in IMOD.

### Plant growth

*Arabidopsis thaliana* Col-0 strain was grown from sterilized seedlings on sterilized plant MS agar media. Harvested *A. thaliana* seeds were sterilized in a sterile fume hood by incubation in 10% bleach for 30 min, washed with sterile water, and then incubated in 70% ethanol for 5 min. The seeds were then washed 6X with sterile water, placed on sterile filter paper, and placed in a dark 4 °C fridge for 3–4 days in a sterile and parafilm-sealed petri dish. Plant agar media plates were prepared by autoclaving Murashige and Skoog (MS) media (0.22% MS media with vitamins, 1.2% plant agar, 0.5% sucrose, pH 5.8) and pouring 100 ml of the media into square petri dishes with 12 cm length. The plates were allowed to cool for 1 h in a sterile fume hood. *A. thaliana* seeds were manually placed on the surface of the plates 1 cm apart by picking up the seeds with sterilized wooden picks. The plates were sealed with parafilm and placed in a climate-controlled plant growth chamber at a 20° angle so the plant roots grew on the surface of the media. The plant chamber was kept at 21 °C with a 16-h light cycle. The plants were allowed to grow for 1 month before use in chemoattraction assays (below).

### Chemotaxis attractant assays with plant roots

Chemoattraction of *Bs* cells to plant roots in the presence and absence of *Sc* spores was conducted on minimal media plates with 0.25% agar. The media was prepared according to previous methods [53] in round petri dishes with 9 cm diameter. One month old sterile *A. thaliana* plants were removed from their sterile media and placed on the edge of the minimal media plates. In total, 3 μl of *B. subtilis* culture was placed to the center of the plate, and then 3 μl of the isolated spore stock was also added to the center. Controls of each bacteria by itself were also prepared. The plates were incubated for 16 h at 30 °C and then placed in a climate-controlled plant growth chamber for 2 weeks. The plant chamber was kept at 21 °C with a 16-h light cycle. After *Sc* colonies were visible, the plates were imaged on a light box.

## Supplementary information

Supplemental Figures

Movie S1

Movie 1

Movie 2

Movie S2
