# Supplementary figures and images for: Microbial hitchhiking: how Streptomyces spores are transported by motile soil bacteria
Source: ISME J. 2021 Mar 15;15(9):2591–600. doi: 10.1038/s41396-021-00952-8 (PMC8397704; doi:10.1038/s41396-021-00952-8)

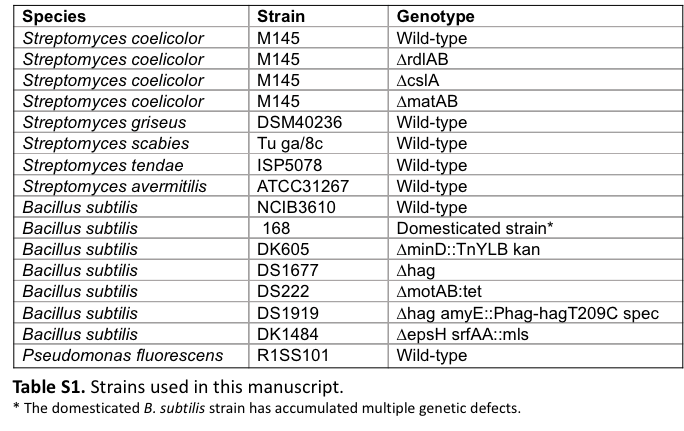


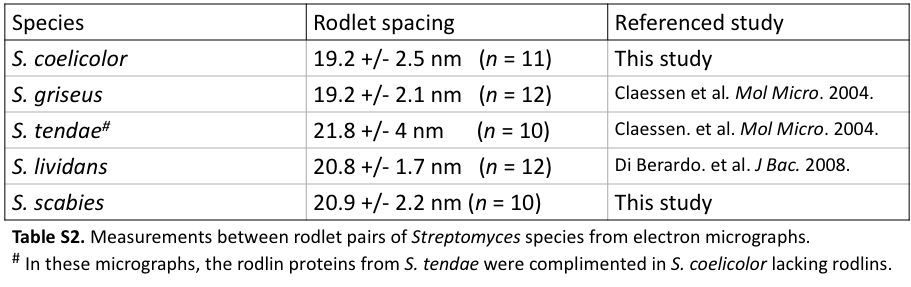


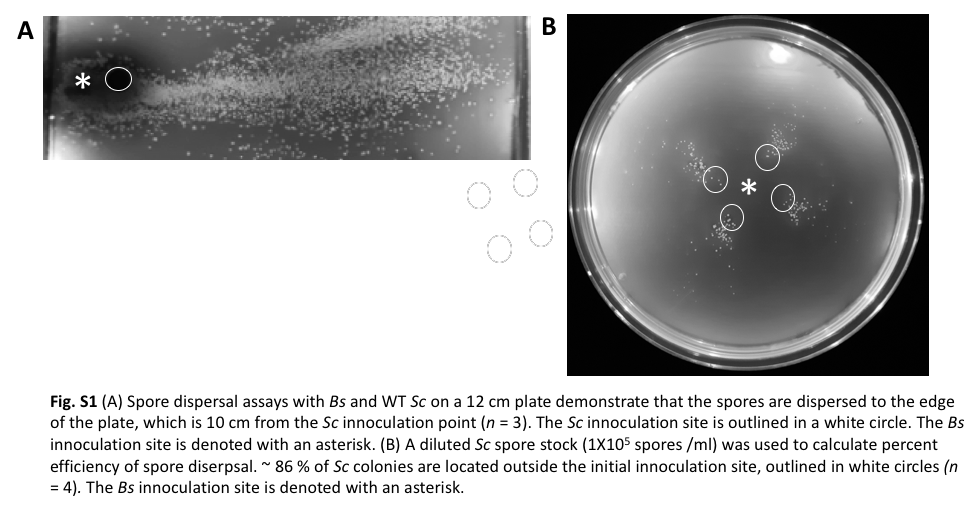


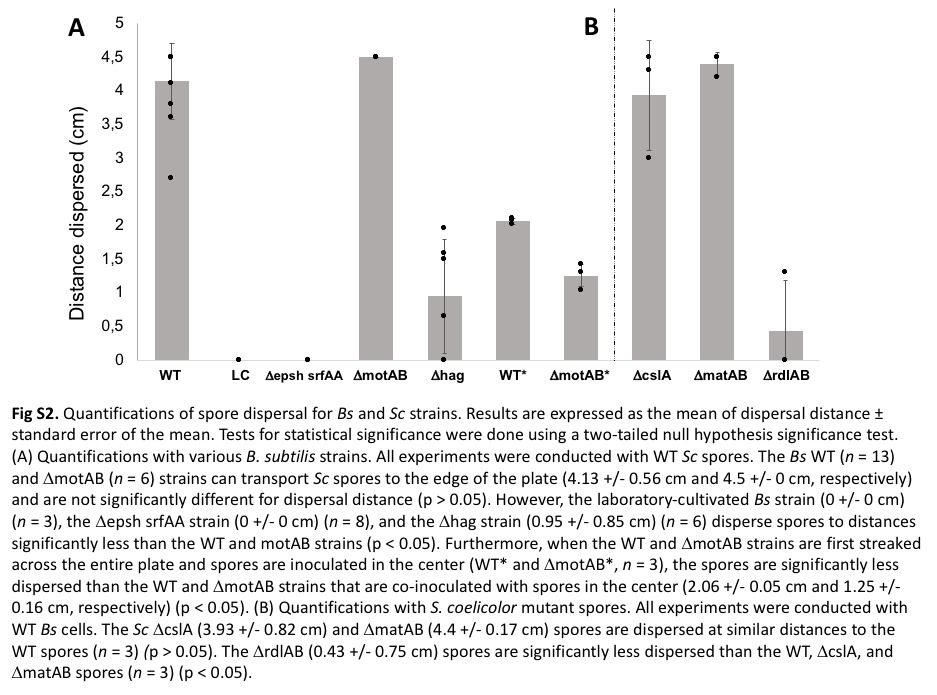


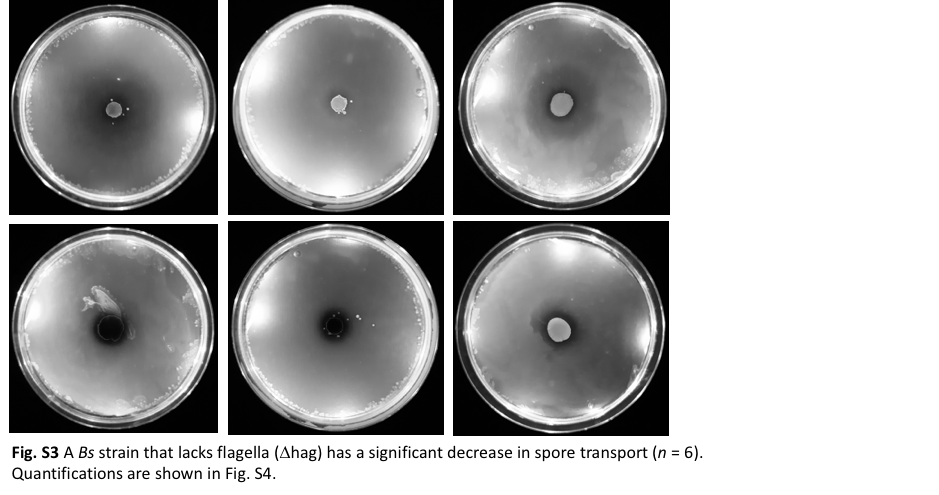


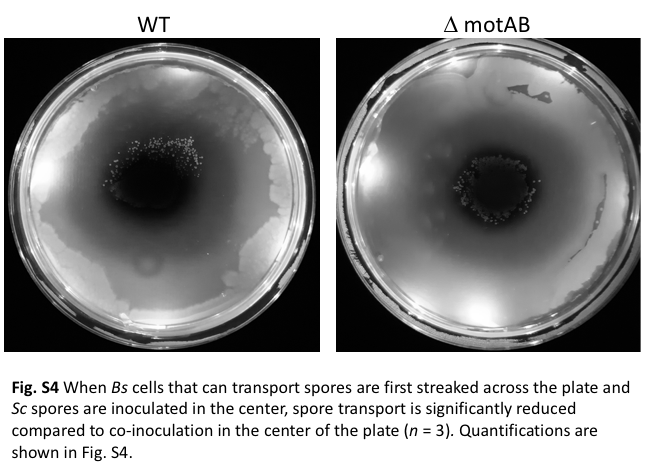


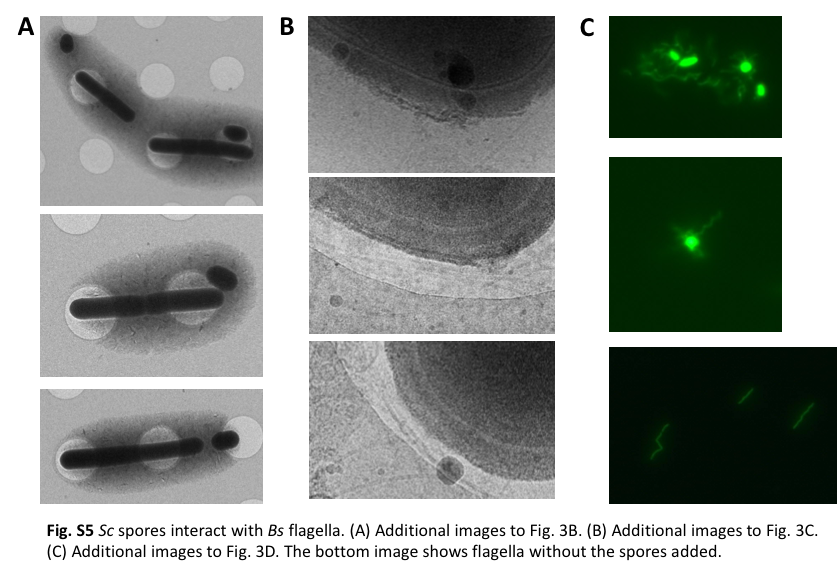


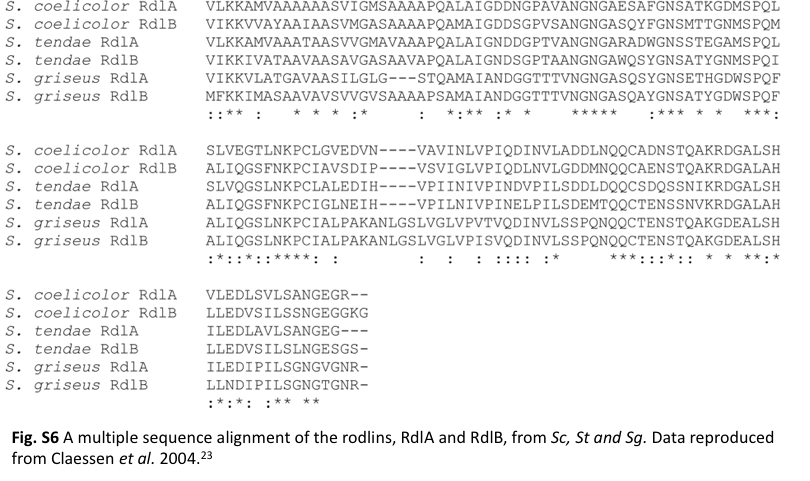


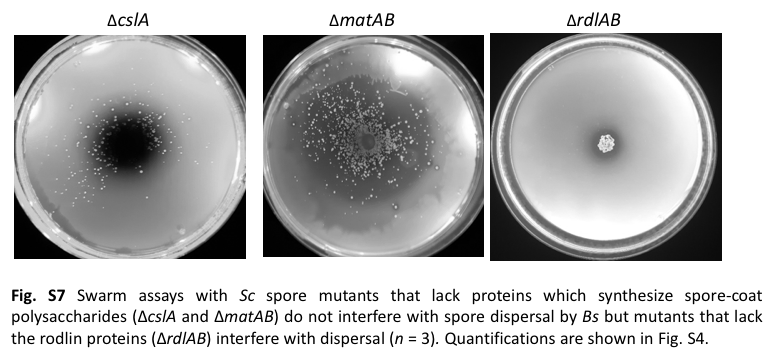


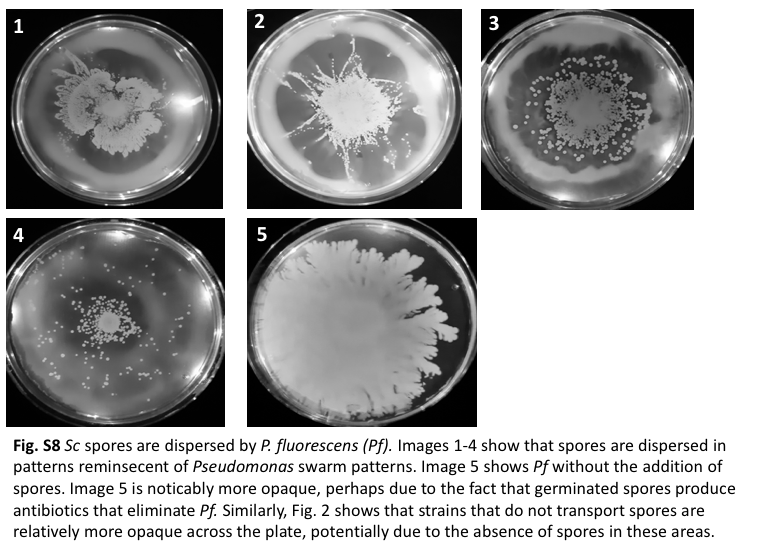

Supplement: Supplementary file 1 — Supplemental Figures [file 41396_2021_952_MOESM1_ESM.docx]
